# All Binder-Free Electrodes for High-Performance Wearable Aqueous Rechargeable Sodium-Ion Batteries

**DOI:** 10.1007/s40820-019-0332-7

**Published:** 2019-11-20

**Authors:** Bing He, Ping Man, Qichong Zhang, Huili Fu, Zhenyu Zhou, Chaowei Li, Qiulong Li, Lei Wei, Yagang Yao

**Affiliations:** 1 Division of Advanced Nanomaterials, Key Laboratory of Nanodevices and Applications, Joint Key Laboratory of Functional Nanomaterials and Devices, CAS Center for Excellence in Nanoscience, Suzhou Institute of Nano-Tech and Nano-Bionics, Chinese Academy of Sciences, Suzhou, 215123 People’s Republic of China; 2 National Laboratory of Solid State Microstructures, College of Engineering and Applied Sciences, Jiangsu Key Laboratory of Artificial Functional Materials, and Collaborative Innovation Center of Advanced Microstructures, Nanjing University, Nanjing, 210093 People’s Republic of China; 3 School of Nano Technology and Nano Bionics, University of Science and Technology of China, Hefei, 230026 People’s Republic of China; 4 Division of Nanomaterials, Suzhou Institute of Nano-Tech and Nano-Bionics, Nanchang, Chinese Academy of Sciences, Nanchang, 330200 People’s Republic of China; 5 School of Electrical and Electronic Engineering, Nanyang Technological University, 50 Nanyang Avenue, Jurong West, 639798 Singapore

**Keywords:** Carbon nanotube fiber, Binder-free electrode, Flexibility, Aqueous rechargeable energy-storage device, Sodium-ion battery

## Abstract

Extensive efforts have recently been devoted to the construction of aqueous rechargeable sodium-ion batteries (ARSIBs) for large-scale energy-storage applications due to their desired properties of abundant sodium resources and inherently safer aqueous electrolytes. However, it is still a significant challenge to develop highly flexible ARSIBs ascribing to the lack of flexible electrode materials. In this work, nanocube-like KNiFe(CN)_6_ (KNHCF) and rugby ball-like NaTi_2_(PO_4_)_3_ (NTP) are grown on carbon nanotube fibers via simple and mild methods as the flexible binder-free cathode (KNHCF@CNTF) and anode (NTP@CNTF), respectively. Taking advantage of their high conductivity, fast charge transport paths, and large accessible surface area, the as-fabricated binder-free electrodes display admirable electrochemical performance. Inspired by the remarkable flexibility of the binder-free electrodes and the synergy of KNHCF@CNTF and NTP@CNTF, a high-performance quasi-solid-state fiber-shaped ARSIB (FARSIB) is successfully assembled for the first time. Significantly, the as-assembled FARSIB possesses a high capacity of 34.21 mAh cm^−3^ and impressive energy density of 39.32 mWh cm^−3^. More encouragingly, our FARSIB delivers superior mechanical flexibility with only 5.7% of initial capacity loss after bending at 90° for over 3000 cycles. Thus, this work opens up an avenue to design ultraflexible ARSIBs based on all binder-free electrodes for powering wearable and portable electronics.

**Electronic supplementary material:**

The online version of this article (10.1007/s40820-019-0332-7) contains supplementary material, which is available to authorized users.

## Highlights

- Nanocube-like KNiFe(CN)_6_ and rugby ball-like NaTi_2_(PO_4_)_3_ are grown on carbon nanotube fibers via simple and mild methods.
- A quasi-solid-state fiber-shaped aqueous rechargeable sodium-ion battery based on all binder-free electrodes is successfully assembled for the first time, delivering a high volumetric capacity of 34.21 mAh cm^−3^ and impressive volumetric energy density of 39.32 mWh cm^−3^.
- The device delivers superior mechanical flexibility with only 5.7% of initial capacity loss after bending at 90° for over 3000 cycles.

**Electronic supplementary material:**

The online version of this article (10.1007/s40820-019-0332-7) contains supplementary material, which is available to authorized users.

## Graphical abstract

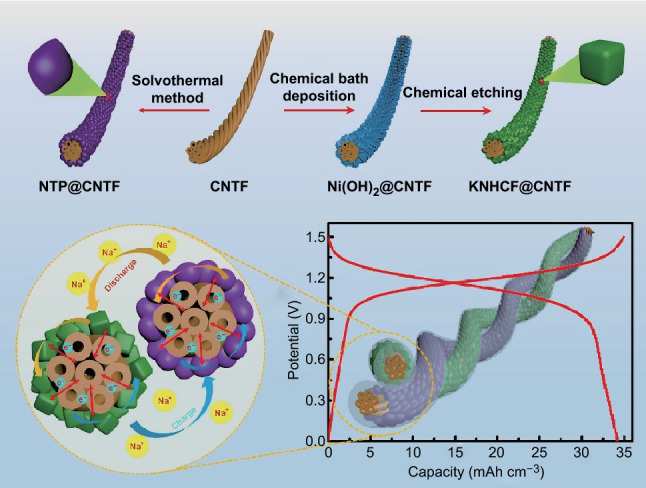

## Introduction

The ever-increasing demand for next-generation wearable electrical products has sparked a boom in the development of matched flexible energy-storage devices with high safety, lightweight, and remarkable biocompatibility [1–15]. In the past decade, sodium-ion batteries (SIBs) have emerged as a promising alternative to lithium-ion batteries, with which they share similar physicochemical properties, for large-scale electricity storage applications due to their abundant resources and low cost [16–21]. However, the conventional SIBs using flammable and toxic organic electrolytes are not suitable as wearable energy-storage devices [5, 22]. Encouragingly, aqueous electrolytes offer the significant advantages of low cost, high ion conductivity, and outstanding safety. It is thus stimulating intense studies on the development of aqueous rechargeable SIBs [5, 23–27]. Huang et al. developed an aqueous rechargeable SIB using flower-like K_0.27_MnO_2_ as the cathode and NaTi_2_(PO_4_)_3_ (NTP) as the anode, exhibiting remarkable specific capacity and rate capacity [23]. Goodenough et al. reported a symmetric SIB with the sodium superionic conductor (NASICON)-structured Na_3_MnTi(PO_4_)_3_ as both the anode and the cathode in an aqueous electrolyte, and it achieved a well-defined voltage plateau of about 1.4 V and excellent performance stability [24]. Very recently, Peng et al. firstly assembled flexible fiber-shaped ARSIBs (FARSIBs) (Na_0.44_MnO_2_//NTP@C) with Na^+^-containing aqueous electrolytes, which delivered remarkable energy density and power density as well as good flexibility [5]. Despite the great progress achieved, all previously reported ARSIBs are fabricated based on powder electrode materials, whose electrode preparation is employed conventional slurry-casting techniques, thus leading to poor flexibility and inferior electrochemical performance [28, 29]. Note that the construction of self-standing active materials has been demonstrated as an effective way to develop high-performance binder-free electrodes for energy-storage devices, which could provide excellent physical and chemical properties and strong adhesion between active materials and substrates for remarkable flexibility [30–32]. Nevertheless, there are few reports focusing on the preparation of binder-free electrodes for ARSIBs, which is attributed to the fact that the most common electrode materials for ARSIBs, such as M_x_MnO_2_ (M = K, Na), Prussian blue and its analogues (PBAs), and NTP, are unsuitable for direct growth on the collectors due to their preparation methods, including high-temperature solid-state, ball-milling, and coprecipitation methods [5, 23, 33–35]. Thus, developing effective and mild methods to fabricate binder-free cathodes and anodes is necessary for high-performance flexible ARSIBs.

PBAs have drawn considerable attention as advanced cathode materials for SIBs due to their open framework structure and large interstitial sites [33, 34, 36–39]. These features are extremely conducive to the intercalation and deintercalation of alkali cations with large ionic radius. However, it is difficult to form strong adhesion between the PBAs and current collectors by traditional coprecipitation methods. To address this problem, we adopt the chemical etching method to grow KNiFe(CN)_6_ (KNHCF) nanocubes on the surface of carbon nanotube fibers (CNTFs) by using nickel hydroxide as a nickel source, directly serving as the cathode (KNHCF@CNTF) for aqueous SIBs. As expected, the KNHCF@CNTF delivers a high volumetric capacity of 58.54 mAh cm^−3^ at 0.05 A cm^−3^ and outstanding rate performance (72.7% retention after a 100-fold increase in current density), demonstrating a superfast charge–discharge characteristic.

Among various anode materials for SIBs, NTP, as a member of the NASICON family with an open framework and remarkable ionic conductivity, is considered as one of the most promising anode materials for SIBs because of its low cost and high theoretical capacity [40–45]. Despite these advantages, the rate performance of NTP is limited by its poor electronic conductivity. High-temperature carbon coating, adopted in most of the previously reported studies, has been regarded as an effective method to overcome this issue [40, 41]. Unfortunately, this method is complex and involves harsh conditions. Herein, we synthesized rugby ball-shaped NTP on CNTFs by a one-step hydrothermal method and directly used them as electrodes (NTP@CNTF), exhibiting a high volumetric capacity (98.4 mAh cm^−3^ at 0.2 A cm^−3^) and excellent rate performance (82.5% retention after a 40-fold increase in current density).

As a proof-of-concept demonstration, we employ KNHCF@CNTF and NTP@CNTF binder-free electrodes to successfully fabricate the first paradigm of a quasi-solid-state FARSIB. Benefiting from the remarkable performance of the binder-free electrodes, the device delivers a high capacity of 34.21 mAh cm^−3^ and impressive energy density of 39.32 mWh cm^−3^.

## Experimental Section

### Preparation of KNHCF@CNTF

KNHCF@CNTF was prepared by a simple chemical bath deposition and subsequent chemical etching process. CNTFs were cleaned with ethanol and deionized water. After being dried at 70 °C overnight, the CNTFs were pretreated in O_2_ plasma for 8 min at 150 W. Firstly, 10 g of NiSO_4_·6H_2_O and 2 g K_2_S_2_O_8_ were dissolved in 100 mL of H_2_O under magnetic stirring and 5 mL aqueous ammonia (28%) was added into the above solution. Then, the pretreated CNTFs were suspended into the reaction for 40 min under slowly stirring. After that, the obtained CNTFs were washed repeatedly with distilled water and dried at 60 °C to get Ni(OH)_2_@CNTFs. Next, the obtained Ni(OH)_2_@CNTFs were placed in a solution of potassium ferricyanide at a certain concentration (1.5, 3.0, and 6.0 mM) for 24 h at room temperature. Finally, the KNHCF@CNTFs were cleaned with deionized water and dried at 60 °C in a vacuum for 12 h. The mass loading of active material on the surface of CNTF (3.0 mM) is about 2.42 mg cm^−2^. For comparison, the powder of KNHCF was obtained by the same method. Ni(OH)_2_ powder (100 mg) and excess potassium ferricyanide were mixed under continuous magnetic stirring at room temperature. After 24 h, the reaction products were collected by centrifugation and washed several times with deionized water. Finally, the KNHCF powder was obtained after vacuum drying at 60 °C overnight.

### Preparation of NTP@CNTF

NTP@CNTF was prepared by a simple and mild solvothermal method. CNTFs were cleaned and dried as described above. Firstly, 1 mL of TiCl_3_ solution (30 wt% HCl) was dropwise added into 30 mL of ethylene glycol under string and kept stirring for 0.5 h. Then, 1 mL of phosphoric acid was dropwise added into the above solution. Next, 1 g of NaH_2_PO_4_·2H_2_O was added into it. After stirring for 0.5 h, the reaction solution and pretreated CNTFs were placed into a 50-mL reaction vessel to heat at 150 °C for 6 h. Finally, the obtained NTP@CNTFs were rinsed with ethanol and distilled water and dried at 80 °C for 24 h. The mass loading of active material on the surface of CNTF is about 2.7 mg cm^−2^. For comparison, the powder product in Teflon-lined stainless autoclave was collected by centrifugation and washed several times with deionized water and ethanol. After dried at 60 °C for 12 h, 0.1 g of obtained powder was added into 50 mL of glucose solution (0.04 M) under magnetic stirring. After stirring for 24 h, the glucose-coated NTP powders were collected by centrifugation and dried at 60 °C overnight. Finally, the NTP@C was obtained after annealing at 600 °C for 4 h in air atmosphere.

## Results and Discussion

**Fig. 1:**
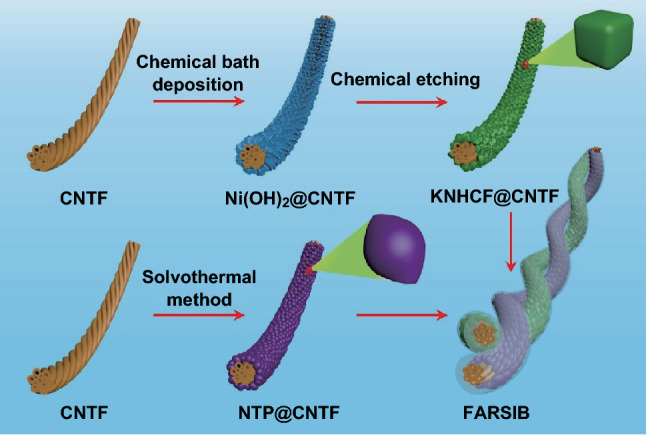
Schematic illustration of the fabrication process of the quasi-solid-state FARSIB

The fabrication procedure of the quasi-solid-state FARSIB is schematically represented in Fig. 1. First, KNHCF nanocubes were synthesized on the surface of CNTFs directly as a binder-free cathode via chemical bath deposition and subsequent chemical etching. The specific reaction process is explained in Scheme S1. Simultaneously, the rugby ball-shaped NTP was grown on CNTFs by a mild solvothermal reaction to serve as an advanced anode for the FARSIB. Thereafter, both the as-fabricated KNHCF@CNTF and NTP@CNTF were immersed in a gel electrolyte consisting of Na_2_SO_4_ and carboxymethyl cellulose sodium (CMC) for a period of time and dried to form a thin layer of gel electrolyte. Finally, the quasi-solid-state FARSIB was fabricated by twisting the cathode and anode together.

### Morphology and Structure of the Cathode Materials

**Fig. 2:**
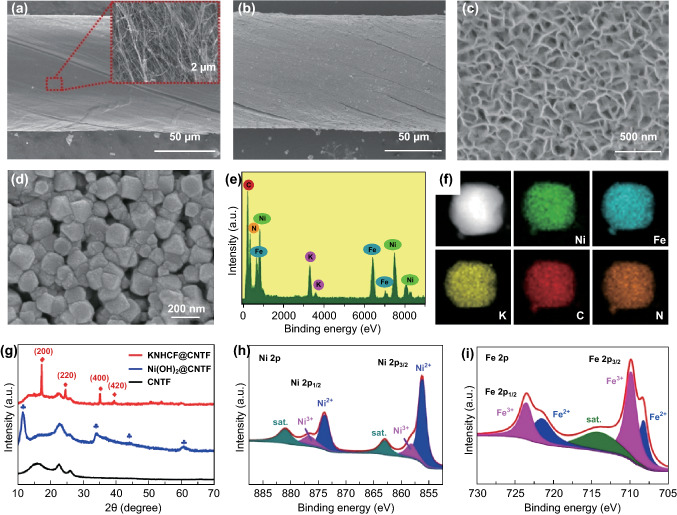
SEM images of **a** pristine CNTF, **b, c** Ni(OH)_2_@CNTF, and **d** KNHCF@CNTF. **e** EDS spectrum of KNHCF cube and **f** corresponding elemental mappings. **g** XRD patterns of KNHCF@CNTF, Ni(OH)_2_@CNTF, and pristine CNTF; XPS survey scan of **h** Ni 2p and **i** Fe 2p

The scanning electron microscopy (SEM) image (Fig. 2a) exhibits a CNTF with a uniform diameter of about 100 μm, which is composed of intertwined carbon nanotubes. In Fig. 2b, c, the SEM images show that Ni(OH)_2_ nanosheet arrays were uniformly coated on the surface of the CNTF by the simple chemical bath deposition method. After chemical etching, the nanosheet arrays are transformed into nanocubes with large porous gaps (Fig. 2d). The internal free space can not only promote the diffusion of electrolyte ions for better rate performance, but also accommodate the volume change of the electrode materials during intercalation and deintercalation of alkali cations, resulting in long cycle life and high specific capacity. It is noted that the fiber-shaped electrode still maintains an admirable tensile strength of about 300 MPa after the active materials are coated on its surface (Fig. S1), which is of great significance for the application of fiber-shaped energy-storage devices. The morphology of a nanocube with a side length of about 160 nm was further investigated by low-resolution transmission electron microscopy (TEM), as displayed in Fig. S2. Figure 2e, f exhibits the energy-dispersive spectroscopy (EDS) spectrum and elemental mapping, suggesting the coexistence and homogeneous distribution of the elements K, Ni, Fe, C, and N. To study the effect of the concentration of potassium ferricyanide solution on morphology, SEM images of the corresponding products at various concentrations are shown in Fig. S3. As the concentration of the reaction solution increases, the nanocubes gradually tend toward regular cube shapes, whereas the free space between the cubes gradually decreases.

More evidence for the conversion of Ni(OH)_2_ to KNHCF was obtained by X-ray diffraction (XRD). As shown in Fig. 2g, the characteristic peaks of the obtained nanosheets on CNTFs can all be indexed to those of α-Ni(OH)_2_ (JCPDS No. 38-0715) except for the background peaks of the CNTFs themselves. After chemical etching, the characteristic peaks located at 17.3°, 24.5°, 35.0°, and 39.3° are consistent with the (200), (220), (400), and (420) phases of KNHCF, while the peaks of Ni(OH)_2_ disappear, implying that the Ni(OH)_2_ nanosheets are converted to KNHCF nanocubes. Figure S4 exhibits the X-ray photoelectron spectroscopy (XPS) survey spectra, further demonstrating the coexistence of the elements K, Ni, Fe, C, and N. The high-resolution Ni 2p XPS spectrum (Fig. 2h) exhibits two main peaks located at 856.4 and 874.0 eV, respectively, consistent with the Ni 2p_3/1_ and Ni 2p_1/2_ states of Ni^2+^, respectively [37]. In addition, the Fe 2p spectrum (Fig. 2i) of KNHCF contains two main peaks located at 709.8 and 723.6 eV, corresponding to those previously reported for Fe^3+^ [46].

### Electrochemical Characterization of the Cathode Materials

**Fig. 3:**
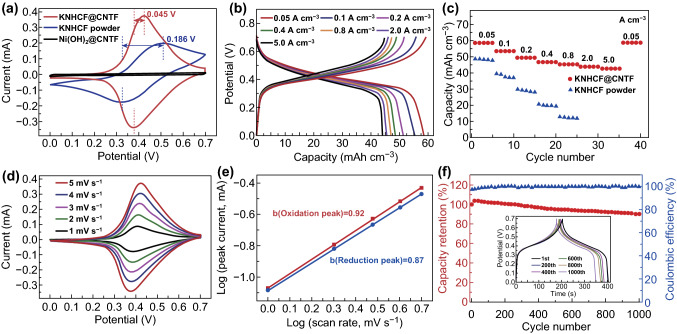
**a** Comparison of CV curves of Ni(OH)_2_@CNTF, KNHCF powder, and KNHCF@CNTF at a scan rate of 5 mV s^−1^. **b** GCD curves of KNHCF@CNTF at various current densities from 0.05 to 5.0 A cm^−3^. **c** Comparison of rate performance of KNHCF powder and KNHCF@CNTF. **d** CV curves at various scan rates, **e** corresponding *b* value, and **f** long-term cycle stability at 0.8 A cm^−3^ of the KNHCF@CNTF

Benefiting from the absence of conductive additives and binders, the cathode material for the ARSIBs was expected to display more efficient charge transfer and ion diffusion. The galvanostatic charge–discharge (GCD) curves at 0.05 A cm^−3^ of KNHCF@CNTF generated under different concentrations of the reaction solution are shown in Fig. S5, indicating that the volumetric capacity of KNHCF@CNTF is the maximum when the concentration is 3.0 mM. Figure 3a displays the cyclic voltammetry (CV) curves of Ni(OH)_2_@CNTF, KNHCF powders, and KNHCF@CNTF at 5 mV s^−1^. Evidently, the Ni(OH)_2_@CNTF exhibits much less coverage than that of KNHCF@CNTF, implying that the untreated Ni(OH)_2_@CNTF is virtually incapable of storing and releasing sodium ions. As revealed in Fig. S6, the pristine CNTF just provides little contribution to the volumetric capacity. In addition, compared with KNHCF powder, the KNHCF@CNTF shows a much lower voltage hysteresis of 0.045 V and higher current, suggesting its faster reaction kinetics and larger capacity, which is further verified by the GCD curves at 0.05 A cm^−3^ (Fig. S7) [11, 47]. As shown in Fig. 3b, the GCD curves at various current densities displayed a stable output voltage plateau of about 0.4 V. It is worth mentioning that the KNHCF@CNTF electrode displays negligible voltage hysteresis at a range from 0.05 to 5.0 A cm^−3^ and only required 21.1 s per discharge or charge at a large current density of 5.0 A cm^−3^, indicating its superfast charge–discharge characteristic. More importantly, benefiting from the fast reaction kinetics, the KNHCF@CNTF electrode achieves a greater volumetric capacity (58.54 mAh cm^−3^ at 0.05 A cm^−3^) and better rate performance (72.7% retention after a 100-fold increase in current density), whereas the KNHCF powder electrode possesses a small volumetric capacity of 48.60 mAh cm^−3^ at the same current density [calculated from the corresponding GCD curves (Fig. S8)] and poor capacity retention of 25.5% when the current density increases to 0.8 A cm^−3^ (Fig. 3c). Such improved rate performance was demonstrated by the electrochemical impedance spectroscopy measurement (Fig. S9). For KNHCF@CNTF, the diameter of the semicircle in the high-frequency region was smaller and the slope of the line in the low-frequency region was greater than that of KNHCF powder, corresponding to better electron conductivity and ion diffusion [11, 48]. This demonstrates that the direct growth of active materials on the conductive substrates is beneficial to the electron transport and ion diffusion. The long-term cyclability of the KNHCF@CNTF was investigated at 0.8 A cm^−3^, and an excellent capacity of 52.85 mAh cm^−3^ was retained after 1000 cycles (Fig. 3f), with a low volumetric capacity decay of only 9.8% and high coulombic efficiency of about 100%, exhibiting outstanding cycle stability and reaction reversibility. The SEM images (Fig. S10) reveal that the free-standing nanocubes with a porous structure are still preserved even after long-term Na^+^ (de)intercalation (500 and 1000 cycles, 0.8 A cm^−3^), further indicating the remarkable structural stability.

Electrochemical kinetic analysis was performed by plotting CV curves to gain further insight into the Na^+^ storage process. Figure 3d shows the CV curves of the KNHCF@CNTF cathode measured at different scan rates from 1.0 to 5.0 mV s^−1^, in which two nearly symmetric redox peaks correspond to the intercalation/extraction of Na^+^ in the KNHCF lattice. The proportion of diffusion-controlled and capacitive contributions can be described by Eq. 1 [49, 50]:

1 $$i = av^{b}$$

where values of *b* close to 0.5 and 1.0 correspond to diffusion-controlled and capacitive behavior, respectively. The values of *b* calculated from the redox peaks are 0.92 and 0.87, respectively, implying a capacitor-like process for the storage of Na^+^. In addition, the percentages of capacitive and diffusion-controlled contributions can be calculated by Eqs. 2 and 3 [51, 52]:

2 $$i = k_{1} v + k_{2} v^{1/2}$$

3 $$i/v^{1/2} = k_{1} v^{1/2} + k_{2}$$

in which *k*_1_ and *k*_2_ are constants. Figure S11 exhibits the percentage contributions of the two kinetic processes at various scan rates. The contributions of the capacitive process are 76.7%, 81.4%, 84.4%, 86.7%, and 87.8% as the scan rates are increased from 1.0 to 5.0 mV s^−1^. Based on the above analysis, the large contribution of capacitive behavior can be responsible for the superfast charge–discharge characteristic and excellent rate performance [50].

To gain insight into the structural evolution of the KNHCF cathode during the charge–discharge process, ex situ XRD and XPS were performed in various states of Na^+^ extraction/insertion (Fig. S12). Clearly, no new characteristic diffraction peaks appear, suggesting that the charge–discharge process belongs to a solid-solution reaction without the formation of a new phase [51]. Moreover, the characteristic (200) and (400) peaks slightly shift to larger angles with the insertion of Na^+^ and returned to their original position after the subsequent charging process, suggesting that the insertion of Na^+^ can lead to a volume decrease in the unit cell. This phenomenon is attributed to the attractive interactions of the inserted Na^+^ with the surrounding C ≡ N groups [53]. In addition, Fig. S13 displays the Na 2p and Fe 2p XPS regions for the fully charged and discharged states. In the Na 2p region, there is no intensity at the initial or the fully charged states, but a peak appears at 1072.3 eV during the discharge process, implying the highly reversible insertion/release of sodium in the host material during the discharge/charge process. To further explore the storage mechanism of sodium ions, the valence states of Fe were measured in various states. Figure S13b shows the Fe 2p XPS regions at the initial state, in which the main peaks at 709.8 and 723.6 eV correspond to the Fe 2p_3/2_ and Fe 2p_1/2_ states of Fe^3+^, respectively. After being discharged to 0 V, the location of the main peak transfers to 708.5 eV, indicating that Fe^3+^ was reduced to Fe^2+^ upon the insertion of sodium ions (Fig. S13a). The main peak then returns to its initial location after the subsequent charging process, further demonstrating the excellent reversibility. Based on the above analysis, the reversible electrochemical reaction of sodium insertion and extraction in the cathode material can be depicted as: *x*Na^+^ + KNi[Fe(CN)_6_] + *x*e^− ^↔ Na_*x*_KNi[Fe(CN)_6_].

### Morphology and Structure of the Anode Materials

**Fig. 4:**
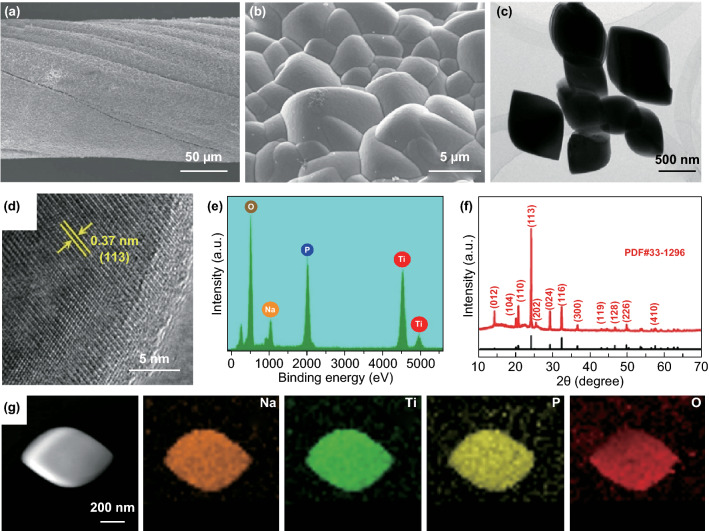
**a**, **b** SEM images of NTP@CNTF with different magnifications. **c, d** TEM images at various magnifications, and **e** EDS spectrum of NTP. **f** XRD pattern of NTP@CNTF. **g** EDS element mappings for Na, Ti, P, and O of NTP

As a high-performance anode material, rugby ball-shaped NTP was synthesized by a one-step solvothermal reaction on CNTFs. The SEM images in Fig. 4a, b show the surface of a CNTF uniformly coated by microparticles. The TEM image (Fig. 4c) reveals that the single active material consisted of rugby ball-shaped micron-scale particles with sizes of about 1 μm. The high-resolution TEM (HRTEM) image (Fig. 4d) reflects the high crystallinity of the rugby ball-shaped NTP, wherein the lattice fringe spacing of 0.37 nm corresponds to its (113) plane. The EDS spectrum (Fig. 4e) and its corresponding EDS mapping images (Fig. 4g) distinctly demonstrate the coexistence and uniform spatial distribution of Na, Ti, P, and O elements. XRD measurement was carried out to further confirm the composition and structure (Fig. 4f), in which all characteristic peaks are indexed to NTP (JCPDS No. 01-085-2265). The successful synthesis of NTP was likewise verified by XPS as shown in Fig. S14, the results of which were consistent with those of XRD and TEM. For comparison, carbon-coated NTP powders (NTP@C) were prepared and investigated by SEM and TEM (Fig. S15). The SEM and TEM images (Fig. S15a, b) show that the NTP@C powder retained the rugby ball-shaped structure. The HRTEM image (Fig. S15c) clearly shows that the NTP surface is uniformly coated with an amorphous carbon shell with a thickness of about 4 nm, forming a core–shell structure.

### Electrochemical Characterization of the Anode Materials

**Fig. 5:**
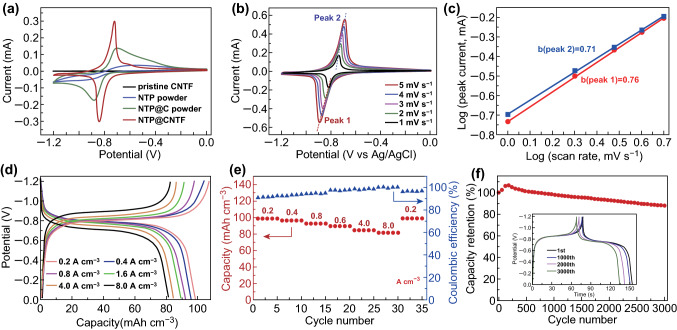
**a** Comparison of the CV curves at 2 mV s^−1^ for NTP@CNTF, CNTF, NTP@C, and NTP powders. **b** CV curves and **c** the corresponding *b* value of NTP@CNTF at various scan rates from 1 to 5 mV s^−1^. **d** GCD curves and **e** rate properties and coulombic efficiency of NTP@CNTF at various current densities from 0.2 to 8.0 mA cm^−3^. **f** Cycle stability of NTP@CNTF at 4.0 A cm^−3^

The electrochemical properties of the obtained NTP@CNTF were investigated in a three-electrode system under the same conditions as for the cathode. Figure 5a compares the CV curves of pristine CNTF, NTP powder, NTP@C powder, and NTP@CNTF measured at 2 mV s^−1^. Like the positive electrode, pristine CNTFs make little contribution to the volumetric capacity. In addition, compared with the NTP and NTP@C powders (same active materials loading with NTP@CNTF), the NTP@CNTF owns smaller separation of the redox peaks and a larger coverage area, indicating its smaller polarization and higher reversible capacity, which is further verified by their GCD curves at 1.6 A cm^−3^ (Fig. S16). As shown in Fig. 5b, the CV curves of NTP@CNTF measured at various scan rates show a pair of sharp redox peaks, corresponding to the characteristic potentials of a Ti^3+^/Ti^4+^ redox couple [54]. Figure 5c shows that the *b* values corresponding to the redox peaks are 0.76 and 0.71, respectively, from which it may be assumed that the sodium storage is a combination of surface-controlled and diffusion-controlled processes [49]. The GCD curves (Fig. 5d) at various current densities demonstrate stable charge–discharge plateaus of about − 0.85/− 0.75 V, which is consistent with the CV results. Besides, Fig. 5e displays the specific capacities of NTP@CNTF at various current densities and its corresponding coulombic efficiency. Remarkably, the NTP@CNTF can achieve an excellent volumetric capacity of 98.4 mAh cm^−3^ at 0.2 A cm^−3^ and possess a high capacity retention of 82.5% as the current density increases to 8.0 mA cm^−3^, suggesting remarkable rate performance. The high coulombic efficiency implies the high reversibility of sodium-ion intercalation and deintercalation at the anode material. The electrochemical impedance spectrum (Fig. S17) indicates the remarkable electronic conductivity and ion diffusivity of the anode. The long-term cycling stability of NTP@CNTF was also explored (Fig. 5f), with 87.6% retention after 3000 cycles at a current density of 4.0 mA cm^−3^. As demonstrated in Fig. S18, the SEM images show that the free-standing rugby ball-shaped structure is well preserved after 1000 cycles and only partly disrupts even after 3000 cycles, suggesting strong adhesion between active material and CNTF. The outstanding electrochemical performance achieved in this work is superior to those of many previously reported NTP-based materials that require high-temperature heat treatment and binders (Table S1).

### Electrochemical Performance and Flexibility Test of as-Prepared FARSIBs

**Fig. 6:**
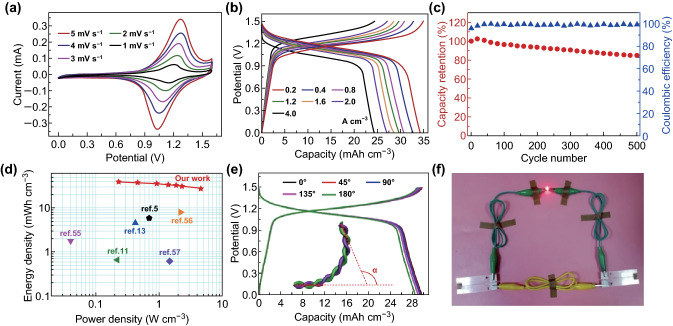
**a** CV curves at various scan rates from 1 to 5 mV s^−1^, **b** GCD curves at various current densities from 0.2 to 4.0 A cm^−3^, **c** cycle stability and corresponding coulombic efficiency at 2.0 A cm^−3^, and **d** Ragone plot of the quasi-solid-state FARSIB with a CMC-Na_2_SO_4_ polymer electrolyte. **e** GCD curves of the FARSIB at various bending angles. **f** LED powered by two FARSIBs in series

Based on the above analysis, an aqueous Na-ion full cell was fabricated using KNHCF@CNTF and NTP@CNTF as the cathode and anode, respectively. To achieve charge balance between them, the length ratio of the cathode and anode is set at about 2:1. Figure S19 shows the corresponding CV curves at 5 mV s^−1^. To verify the potential of the two binder-free electrodes for wearable energy-storage devices, a quasi-solid-state FARSIB was successfully assembled with a CMC-Na_2_SO_4_ polymer electrolyte. A low-resolution SEM image (Fig. S20) of the fiber-shaped device displayed a twisted structure. Figure 6a shows the CV curves of this FARSIB, all of which exhibit a pair of apparent redox peaks at various scan rates from 1 to 5 mV s^−1^. The GCD curves (Fig. 6b) were measured at various current densities from 0.2 to 4.0 A cm^−3^ and displayed a stable discharge plateau of ~ 1.15 V. Notably, the FARSIB delivers a volumetric capacity of 34.21 mAh cm^−3^ at 0.2 A cm^−3^ and can retain a high capacity of 24.23 mAh cm^−3^ at 4.0 A cm^−3^, implying its remarkable rate properties. The long-term cycling stability and corresponding coulombic efficiency of the FARSIB were tested at 2.0 A cm^−3^ (Fig. 6c), still maintaining about 84.7% of initial capacity after 500 cycles and high coulombic efficiency close to 100%. The volumetric energy and power density of our FARSIB are displayed in Fig. 6d. The FARSIB reaches a maximum volumetric energy density of 39.32 mWh cm^−3^ and a maximum volumetric power density of 4.60 W cm^−3^, which considerably exceed the values previously reported for other fiber-shaped energy-storage devices, such as Co//Zn battery [13], GO/MWCNT//MoS_2_-rGO/MWCNT [55], Ni//Zn battery [11], NiCo//Zn battery [56], titanium wire/Co_3_O_4_ nanowires//carbon fibers/graphene [57], and NIB [5]. In addition, based on the total mass of the fiber-shaped cathode and anode (including active and inactive components), the specific energy/power densities were calculated as shown in Fig. S21. Thanks to the lightweight CNTFs, the assembled FARSIB exhibits a maximum specific energy density of about 27.72 Wh kg^−1^ and still maintains a high specific energy density of about 19.61 Wh kg^−1^ achieving a maximum power density of about 3.24 KW kg^−1^. For fiber-shaped energy-storage devices, mechanical flexibility is of great significance for practical application as a power source for wearable electronic devices. As shown in Fig. 6e, there are negligible changes in capacity at 1.2 A cm^−3^ under various bending angles, exhibiting the excellent mechanical flexibility of the assembled device. Furthermore, the full battery exhibits superior mechanical stability with only 5.7% of initial capacity loss after bending at 90° for 3000 cycles (Fig. S22), which can be attributed to the absence of binders and strong adhesion between the active materials and CNTF. Figure S23 exhibits the electrochemical impedance spectroscopies of the assembled FARSIB before and after bending tests, indicating the slight increase in the contact resistance (*R*_s_) and faradic charge-transfer resistance (*R*_ct_) of the electrodes after the bending test. In addition, an LED could be powered by two of the assembled fiber-shaped batteries connected in series (Fig. 6f), and the brightness of the LED barely changed when the devices were bent at various angles (Fig. S24). To further demonstrate the potential of the FARSIBs in wearable electronics, two assembled FARSIBs were woven into the flexible textile with an abbreviation (NB) of “Na-ion battery” and powered for a red LED shown in Fig. S25, verifying the practicability and high flexibility of our FARSIBs.

## Conclusion

In summary, a prototype FARSIB was fabricated for the first time by growing nanocube-like KNHCF and rugby ball-shaped NTP on CNTFs directly as the cathode and anode, respectively. Benefiting from the absence of binders, both the cathode and anode exhibit remarkable electronic conductivity and ionic diffusion, accordingly delivering excellent capacities and rate performance. More encouragingly, the assembled quasi-solid-state fiber-shaped SIB could achieve a high capacity of 34.21 mAh cm^−3^ and impressive energy density of 39.32 mWh cm^−3^. Furthermore, due to the strong adhesion between the active materials and CNTFs, the capacity of our devices barely decayed when bent at a range of angles, displaying admirable mechanical flexibility. Thus, this work may be a stepping stone toward the development of all binder-free electrodes for next-generation wearable ARSIBs.

## Electronic supplementary material

Below is the link to the electronic supplementary material.

Supplementary material 1 (PDF 1395 kb)
