# Supplementary material for: All Binder-Free Electrodes for High-Performance Wearable Aqueous Rechargeable Sodium-Ion Batteries
Source: Nanomicro Lett. 2019 Nov 20;11:101. doi: 10.1007/s40820-019-0332-7 (PMC7770778; doi:10.1007/s40820-019-0332-7)
Supplement: Supplementary file 1 — Supplementary material 1 (PDF 1395 kb) [file 40820_2019_332_MOESM1_ESM.pdf]

Supporting information for

All Binder-Free Electrodes for High-Performance Wearable Aqueous Rechargeable Sodium-Ion Batteries

Bing He[1, 3, 4, +], Ping Man[1, 3, +], Qichong Zhang[5], Huili Fu[1, 3], Zhenyu Zhou[1, 3], Chaowei Li[1, 3], Qiulong Li[1, 2], Lei Wei[5], Yagang Yao[1, 2, 4, *]

[1]Division of Advanced Nanomaterials, Key Laboratory of Nanodevices and Applications, Joint Key Laboratory of Functional Nanomaterials and Devices, CAS Center for Excellence in Nanoscience, Suzhou Institute of Nano-tech and Nano-bionics, Chinese Academy of Sciences, Suzhou 215123, People's Republic of China

[2]National Laboratory of Solid State Microstructures, College of Engineering and Applied Sciences, Jiangsu Key Laboratory of Artificial Functional Materials, and Collaborative Innovation Center of Advanced Microstructures, Nanjing University, Nanjing 210093, People's Republic of China

[3]School of Nano Technology and Nano Bionics, University of Science and Technology of China, Hefei 230026, People's Republic of China

[4]Division of Nanomaterials, Suzhou Institute of Nano-Tech and Nano-Bionics, Nanchang, Chinese Academy of Sciences, Nanchang 330200, People's Republic of China

[5]School of Electrical and Electronic Engineering, Nanyang Technological University, 50 Nanyang Avenue, 639798, Singapore

[+] These authors contributed equally to this work.

*Corresponding author. E-mail: ygyao2018@nju.edu.cn (Yagang Yao)

S1 Experimental Section

Preparation of CNTF: Pristine CNTFs are commercialized products which purchased from Suzhou Creative Nano Carbon Co.,Ltd and the detailed preparation process is as follows. Firstly, a raw CNT aerogel was obtained via injecting a solution of ethyl alcohol containing 1.2 wt% ferrocene and 0.4 wt% thiophene, which was carried by Ar/H_2 into a furnace at 20 mL h^{-1} and took place chemical reactions at a high temperature approximately 1300 °C. Secondly, the raw CNT aerogel was treated via chemical vapor deposition process at 720 °C in a mixed gas including 400 sccm Ar, 400 sccm H_2 and 100 sccm C_2H_2 to acquire a stable and robust CNT

aerogel film. Finally, after the CNT strips shrank through the treatment of ethanol, the CNTF was prepared by quickly spinning the obtained CNT strips.

Assembly of FARSIBs: The as-fabricated FARSIBs were assembled by adopting binder-free KNHCF@CNTF as the cathode, NTP@CNTF as the anode, and Na_2SO_4-carboxymethyl cellulose sodium (CMC) as the gel electrolyte. The Na_2SO_4–CMC gel electrolyte was prepared by mixing 5 g Na_2SO_4 and 3 g CMC in 50 mL distilled water under vigorous stirring at 90 °C for 2 h until the solution became clear. The as-prepared KNHCF@CNTF and NTP@CNTF were immersed in the gel electrolyte for 10 min to allow the active material full contact with the electrolyte and dried at 60 °C for 15 min. Then repeat the process. The FARSIB was assembled by twisting the cathode and anode and dried at 60 °C for 10 h.

S2 Electrochemical Performance Measurements

The electrochemical characterizations of obtained electrode materials was analyzed by cyclic voltammetry curves, galvanostatic charge/discharge curves, and electrochemical impedance spectroscopy measured on an electrochemical workstation (CHI 760E, Chenhua). For three electrode system tests, KNHCF@CNTF or NTP@CNTF was directly used as the working electrode, Pt wire and Ag/AgCl were used as the counter electrode and the reference electrode, respectively, 1 M Na_2SO_4 was used the aqueous electrolyte. For the FARSIB, the KNHCF@CNTF and NTP@CNTF were used as the cathode and anode, respectively, with the Na_2SO_4–carboxymethyl cellulose sodium (CMC) as the gel electrolyte. For comparison, the powder slurry was prepared by mixing the obtained active materials (KNHCF, NTP, and NTP@C), acetylene black, and polytetrafluoroethylene at a weight ratio of 7:2:1 with N-Methyl pyrrolidone as solvent. After stirring for 2 h, repeated dip-coating method was adopted to achieve the uniform coating of the same loading active materials on the surface of CNTF. Finally, the CNTF was dried at 60 °C in vacuum overnight to obtain the corresponding powder electrodes.

S3 Characterizations of Materials

The morphologies and microstructures of the electrodes and pressure sensor were analyzed by using a scanning electron microscope (Hitachi S-4800, 5 KV). The crystal structure and chemical composition of samples were characterized by X-ray diffraction (Rigaku D/MAX2500 V) and X-ray photoelectron spectrometer (ESCALab MKII). Transmission electron microscopy images were measured by a high-resolution transmission electron microscope (FEI Tecnai G2 20). The calculation equation of the electrode volume :

$$V = \frac{\pi D^2 L}{4} \qquad \text{(S1)}$$

Where V (cm^3) represents the total volume of the electrodes, D (cm) is the diameter of the electrodes with electrochemical active materials and L (cm) is the length of the electrodes.

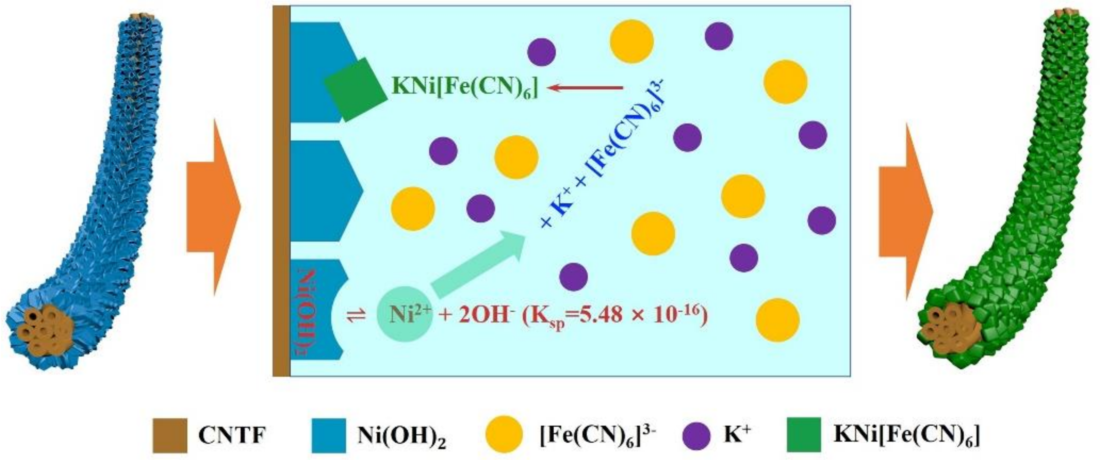

Scheme S1 Schematic representation of the synthetic procedure for the transformation of $Ni(OH)_2$@CNTF into the more stable KNHCF@CNTF

As shown in Scheme S1, $Ni(OH)_2$ nanosheets serve as self-sacrificing template to provide Ni source for the direct growth of KNHCF cubes on the CNTF by a simple chemical etching method, in which Ni^{2+} produced by local dissolution ($Ni(OH)_2 \rightleftharpoons Ni^{2+} + 2(OH)^-$) of $Ni(OH)_2$ nanosheets reacts with $K_3[Fe(CN)_6]$ to obtain the more stable KNHCF cubes. As the reaction time increases, $Ni(OH)_2$@CNTF gradually transforms into KNHCF@CNTF.

S4 Supplementary Figures and Table

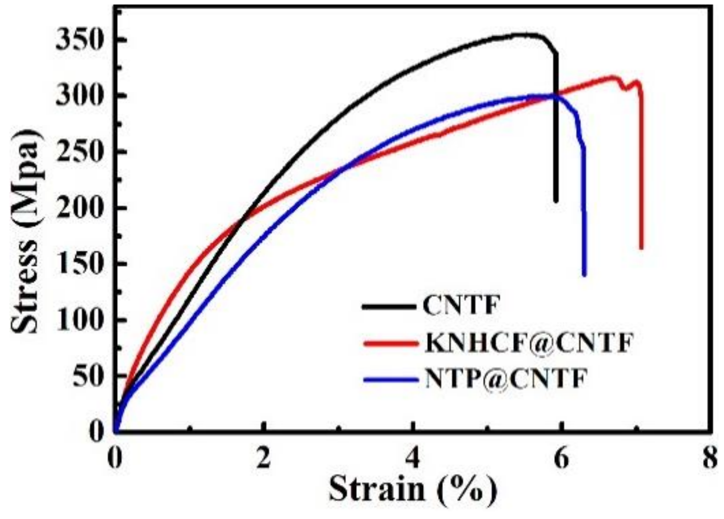

Fig. S1 Tensile strength test of pristine CNTF, NTP@CNTF and KNHCF@CNTF

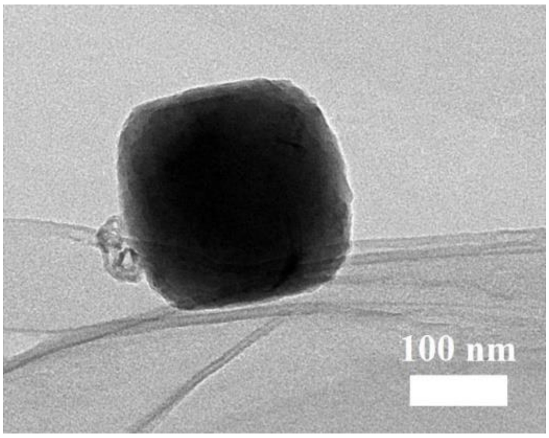

Fig. S2 Low-resolution transmission electron microscopy (TEM) image of KNHCF

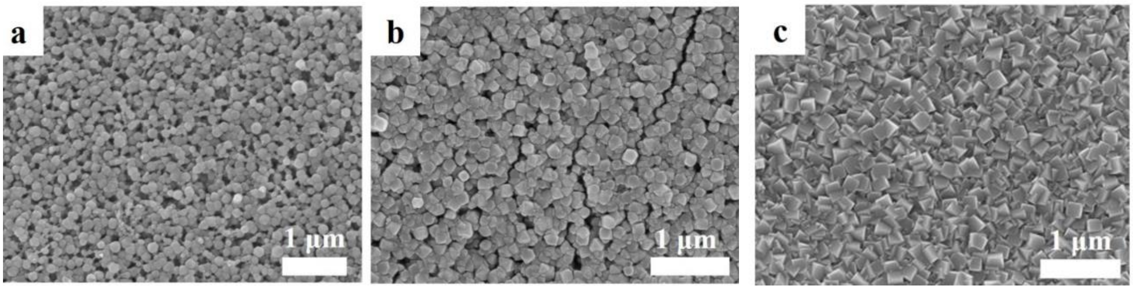

Fig. S3 SEM images of KNHCF cubes with different concentration of the reaction solution : **a** 1.5 mM; **b** 3.0 mM; **c** 6.0 mM

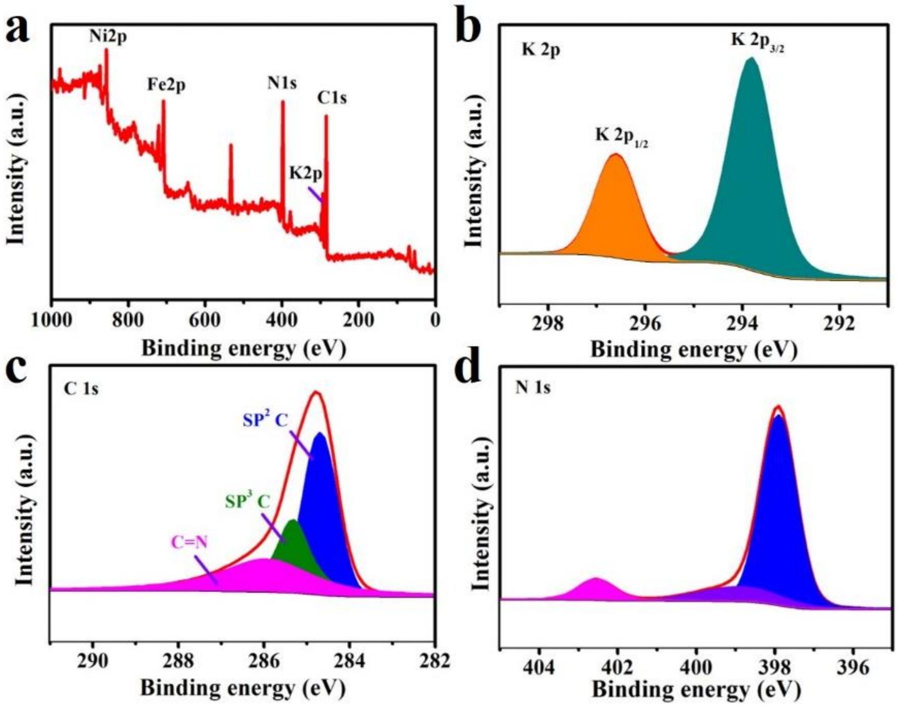

Fig. S4 XPS survey scans of **a** full spectrum, **b** K 2P, **c** C 1s, and **d** N 1s regions for the KNHCF

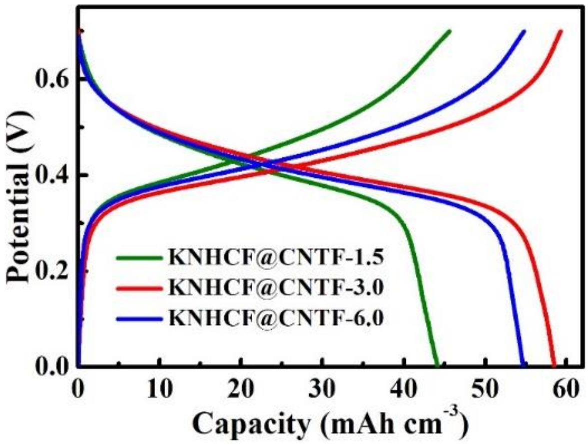

Fig. S5 GCD curves at a current density of 0.05 A cm^{-3} of KNHCF@CNTF generated under different concentrations of the reaction solution

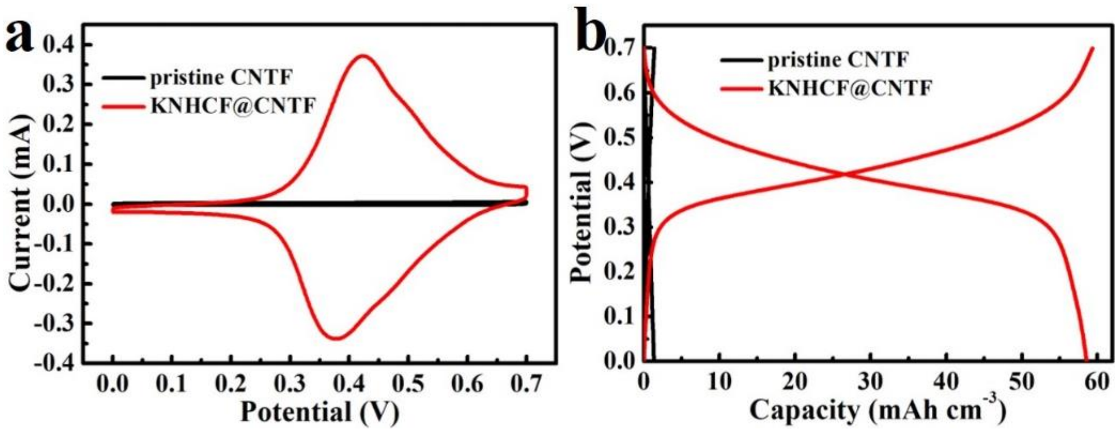

Fig. S6 Comparison of **a** CV curves at a scan rate of 5mV s^{-1} and **b** GCD curves at a current density of 0.05 A cm^{-3} of KNHCF@CNTF and pristine CNTF

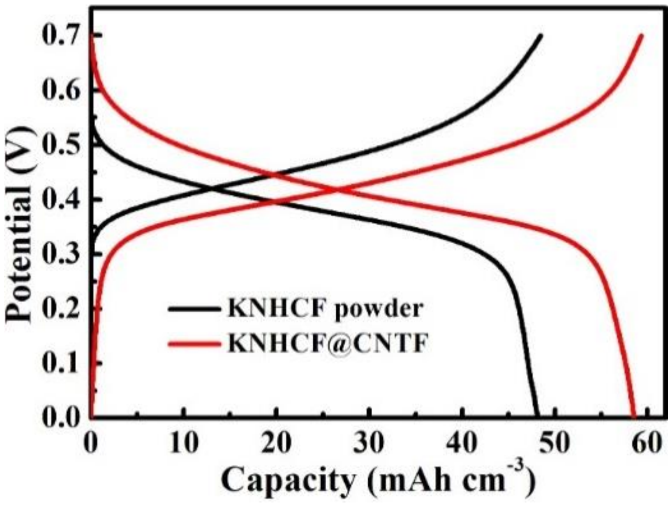

Fig. S7 Comparison of GCD curves of KNHCF@CNTF and KNHCF powder at a current density of 0.05 A cm^{-3}

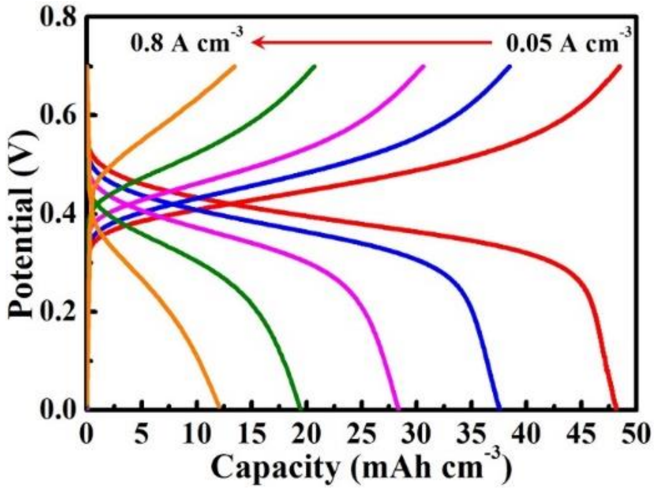

Fig. S8 The GCD curves of the KNHCF powder at various current densities

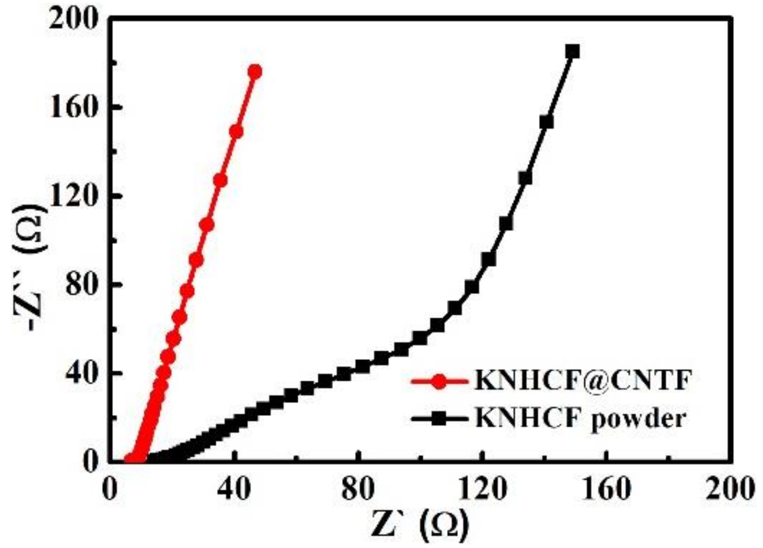

Fig. S9 Electrochemical impedance spectroscopy of KNHCF@CNTF and KNHCF powder

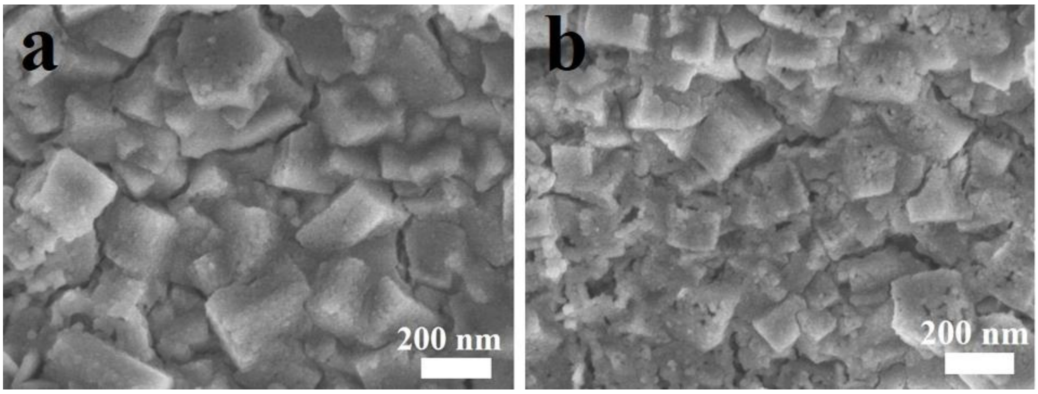

Fig. S10 The SEM images of KNHCF@CNTF after 500 cycles (**a**) and 1000 cycles (**b**)

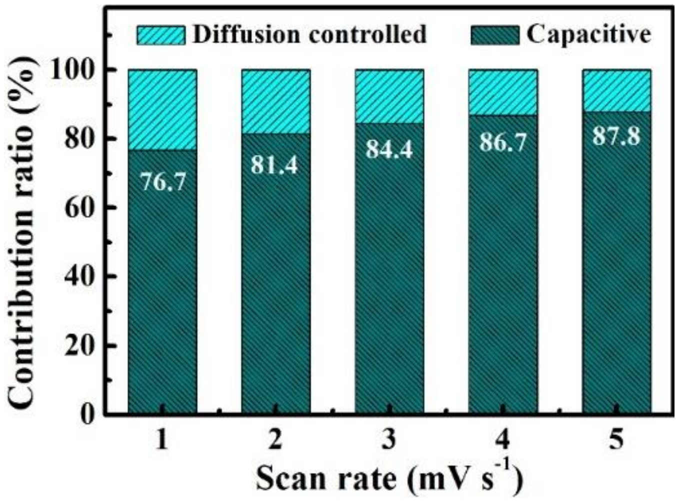

Fig. S11 The contribution ratio at various scan rates of the KNHCF@CNTF

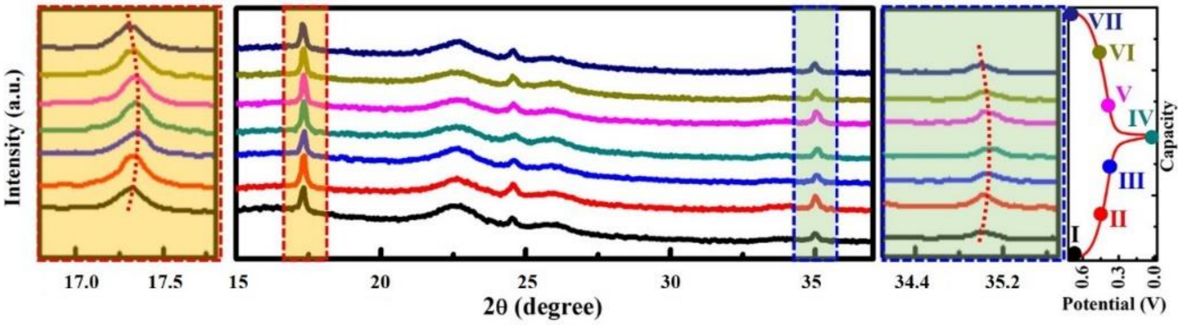

Fig. S12 Ex-situ XRD pattern of KNHCF@CNTF in the different states of Na^+ extraction/insertion

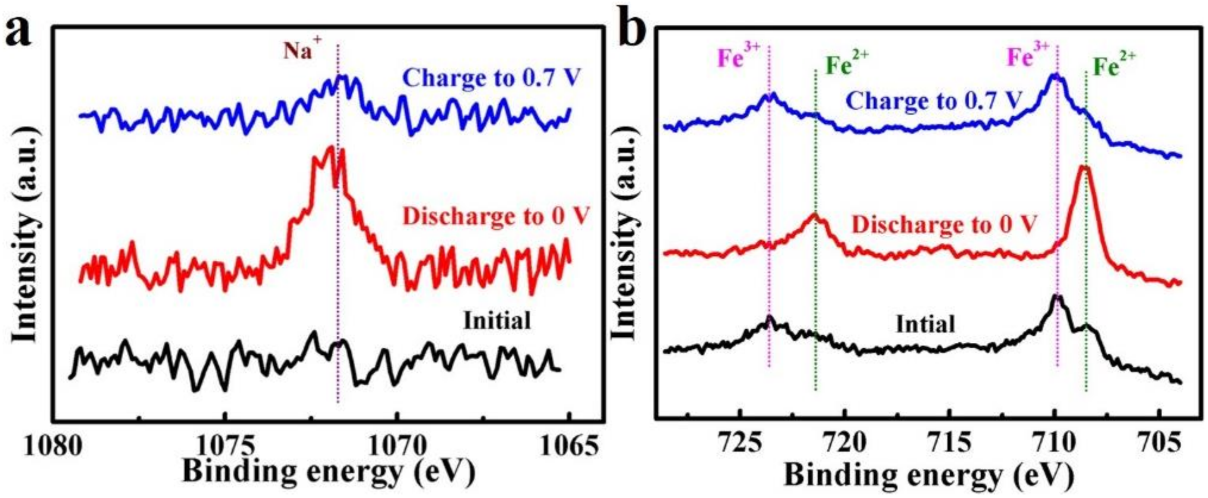

Fig. S13 Ex-situ XPS spectra of Na 1s and Fe 2p at different states

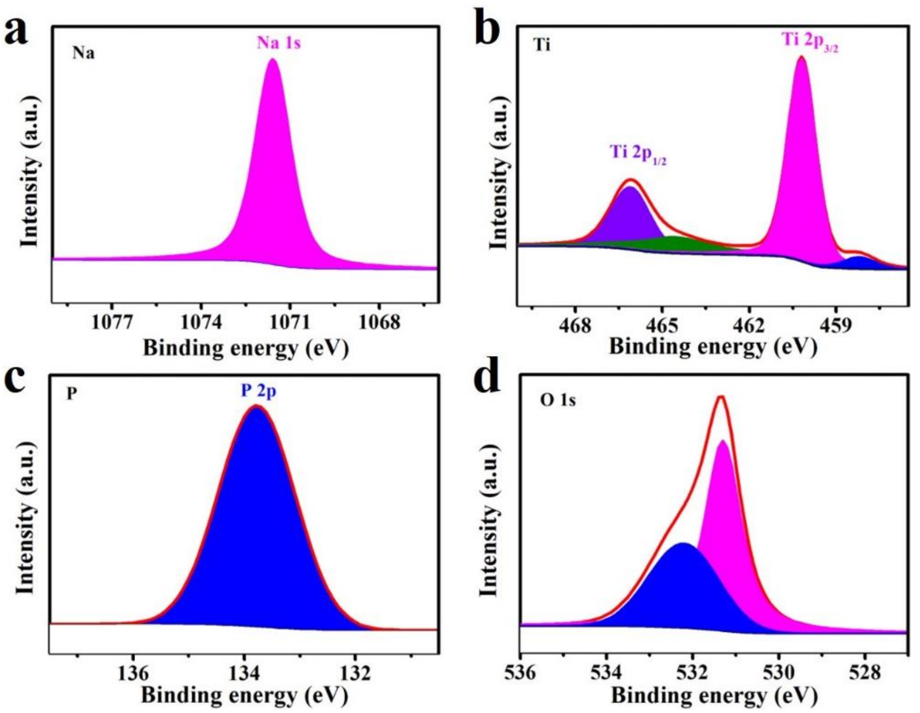

Fig. S14 XPS survey scans of **a** Na 1s, **b** Ti 2p, **c** P 2p, and **d** O 1s regions for the NTP@CNTF

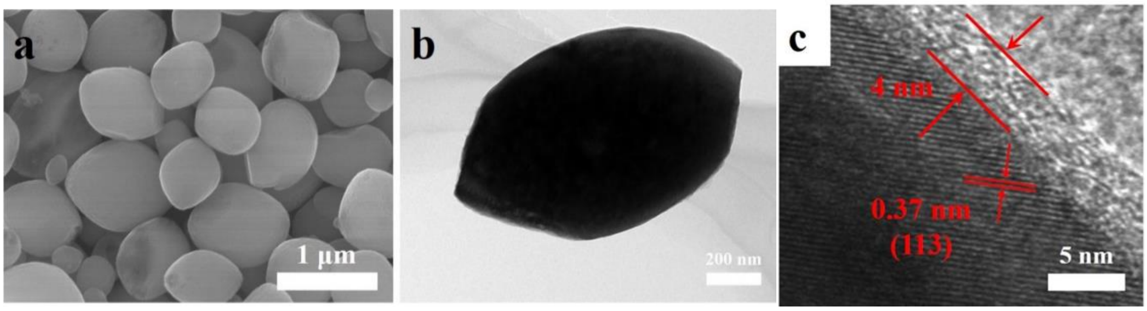

Fig. S15 a SEM image and **b, c** TEM images at different magnifications of NTP@C

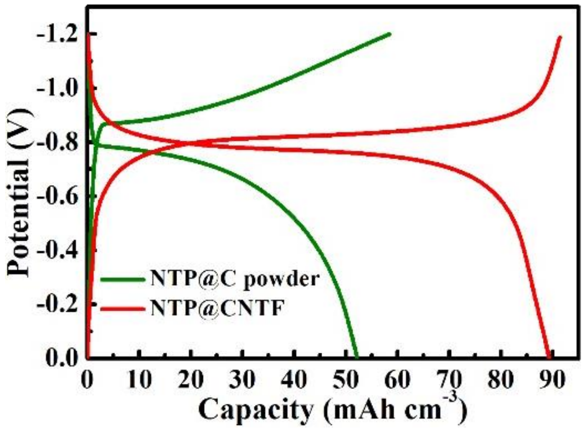

Fig. S16 GCD curves of NTP@CNTF and NTP@C powder at a current density of 1.6 A cm^{-3}

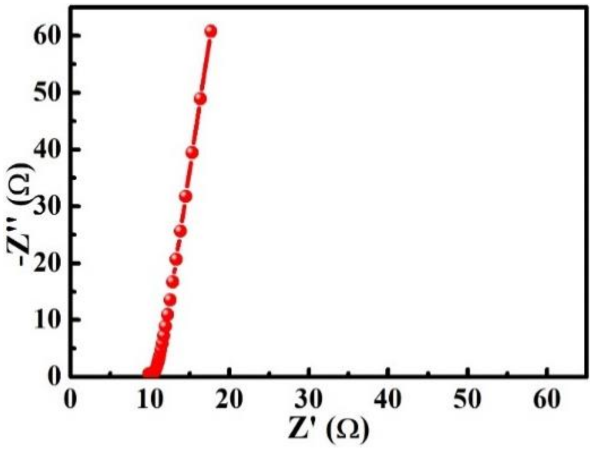

Fig. S17 Electrochemical impedance spectroscopy of NTP@CNTF

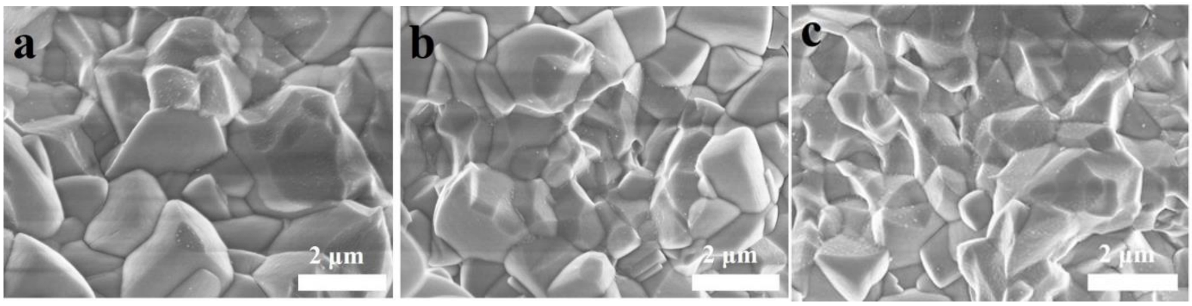

Fig. S18 The SEM images of NTP@CNTF after different charge-discharge cycles: **a** 1000 cycles; **b** 2000 cycles; **c** 3000 cycles

Table S1 Comparison of our anode material (NTP@CNTF) with the NTP-based anode materials in aqueous SIBs reported before

Sample	Annealing temperature	Electrode preparation	Capacity (max)	Capacity (min)	Refs.
**NTP@CNTF**	**No**	**Binder-free**	**98.4 mAh cm^{-3} at 0.2 A cm^{-3} (122 mAh g^{-1} at 0.25 A g^{-1})**	**81.2 mAh cm^{-3} at 8.0 A cm^{-3} (100 mAh g^{-1} at 10.0 A g^{-1})**	**This work**
NTP/C	900°C	Slurry-coasting	75 mAh g^{-1}		[S1]
NTP/C	700°C	Slurry-coasting	100 mAh g^{-1} at 1 C	86 mAh g^{-1} at 10 C	[S2]
NTP/C	800°C	Slurry-coasting	98 mAh g^{-1} at 1 C	48 mAh g^{-1} at 50 C	[S3]
NTP/C	700°C	Slurry-coasting	127 mAh g^{-1} at 1 C	100 mAh g^{-1} at 1 C	[S4]
NTP/MWNTs	700°C	Slurry-coasting	120 mAh g^{-1} at 1 C	80 mAh g^{-1} at 1 C	[S5]
NTP/C	700°C	Slurry-coasting	105 mAh g^{-1} at 0.5 A g^{-1}	80 mAh g^{-1} at 3.0 A g^{-1}	[S6]
NTP/C	700°C	Slurry-coasting	119.4 mAh g^{-1} at 1 C	63 mAh g^{-1} at 50 C	[S7]
NTP/GNS	700°C	Slurry-coasting	100 mAh g^{-1} at 2 C	41.5 mAh g^{-1} at 20 C	[S8]
NTP/TiN	700°C	Slurry-coasting	131 mAh g^{-1} at 2 C	~52 mAh g^{-1} at 10 C	[S9]
NTP/C	800°C	Slurry-coasting	103 mAh g^{-1} at 3 C	72 mAh g^{-1} at 90 C	[S10]

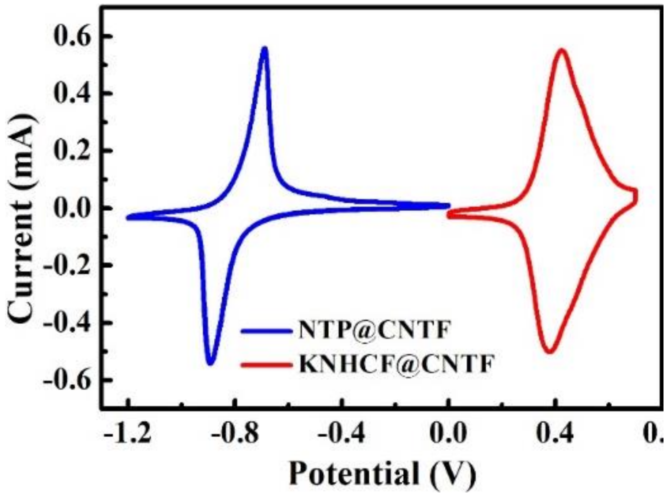

Fig. S19 CV curves at a scan rate of 5 mV s^{-1} of cathode and anode with a length ratio of 2:1.

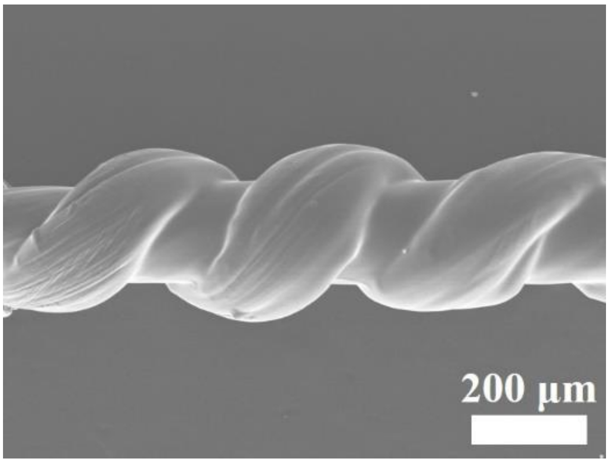

Fig. S20 Low-resolution SEM image of the fiber-shaped SIB

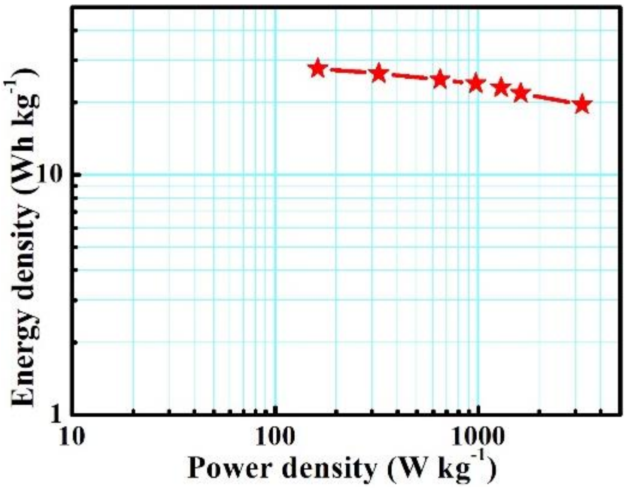

Fig. S21 Specific energy and power densities of the assembled FARSIB

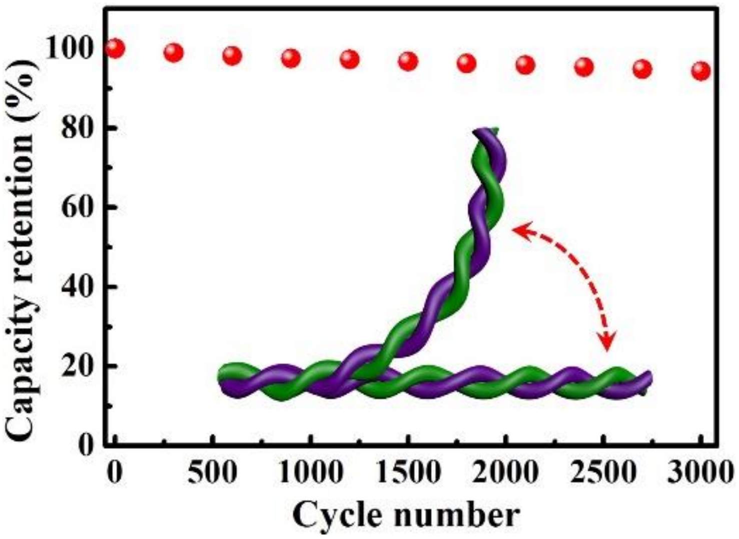

Fig. S22 Normalized capacitances of our fiber-shaped device bent 90° for 3000 cycles

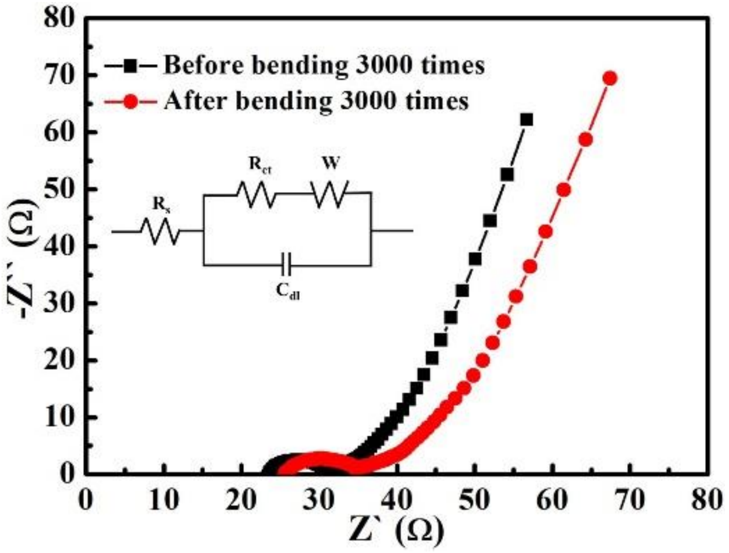

Fig. S23 Electrochemical impedance spectroscopies of the assembled FARSIB before and after bending 3000 times

As shown in **Fig.** S23, the equivalent circuit consists of the series resistance (R_s), double layer capacitance (C_{dl}), charge transfer resistance (R_{ct}) and Warburg behavior (W). R_s represents the total resistance of the electrolyte and electrical contacts, which is estimated by the value of the intercept at the real axis is used. R_{ct} is the faradic charge-transfer resistance at the interface between the electrode and the electrolyte, which is related to the semicircle diameter in the plot. After bending 3000 times, the R_s increases from 23.57 to 25.54 Ω, while the R_{ct} increases from 7.68 to 9.49 Ω, indicating the slight increase of the contact resistance and faradic charge-transfer resistance of the electrodes after the bending test.

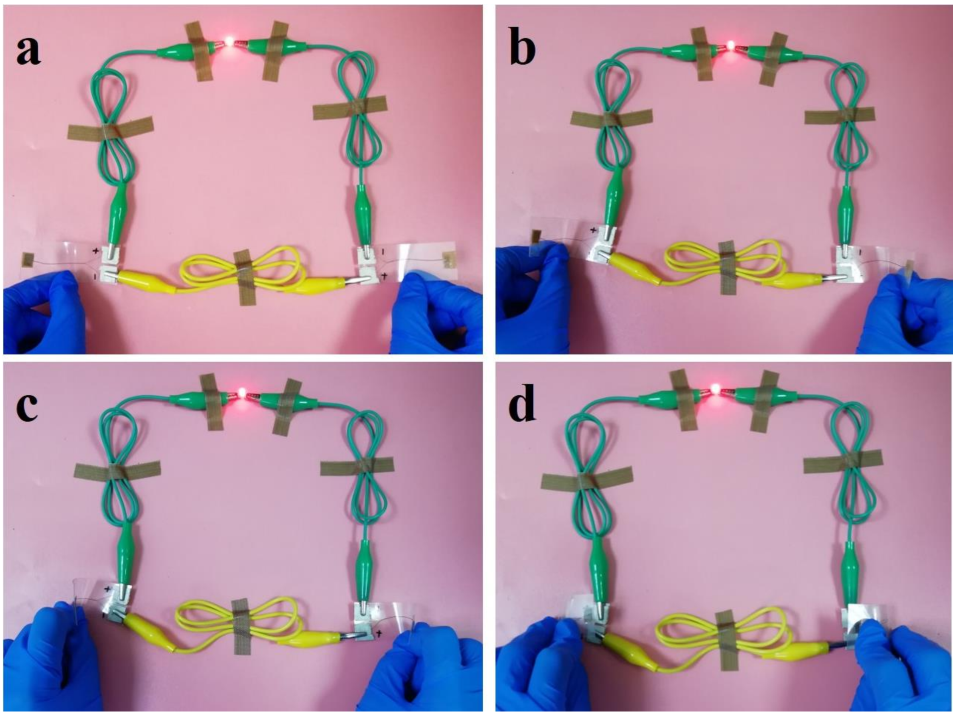

Fig. S24 A LED powered by two FARSIBs in series under different bend angles

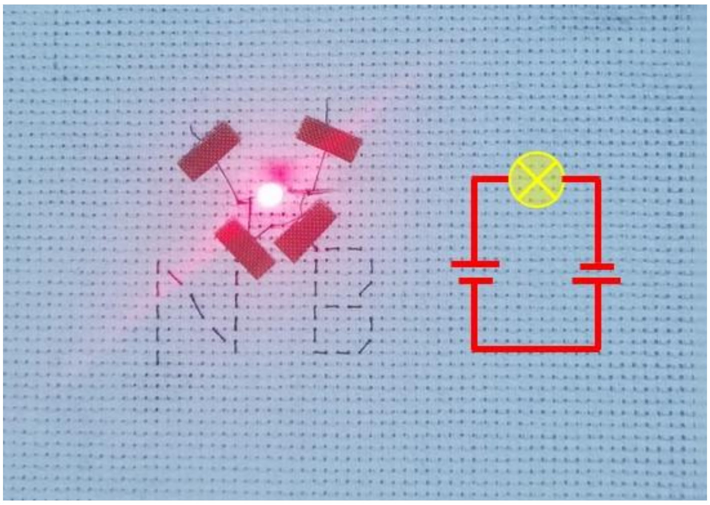

Fig. S25 Photograph of two assembled fiber-shaped batteries woven into flexible textiles and power for a red LED

Supplementary References

[S1]Q. Zhang, C. Liao, T. Zhai, H. Li, A high rate 1.2 V aqueous sodium-ion battery based on all nasicon structured $NaTi_2(PO_4)_3$ and $Na_3V_2(PO_4)_3$. Electrochim. Acta **196**, 470-478 (2016). https://doi.org/10.1016/j.electacta.2016.03.007

[S2]X. Wu, Y. Cao, X. Ai, J. Qian, H. Yang, A low-cost and environmentally benign aqueous

rechargeable sodium-ion battery based on $NaTi_2(PO_4)_3$–$Na_2NiFe(CN)_6$ intercalation chemistry. Electrochem. Commun. **31**, 145-148 (2013). https://doi.org/10.1016/j.elecom.2013.03.013

[S3]X. Cao, L. Wang, J. Chen, J. Zheng, A low-cost Mg^{2+}/Na^{+} hybrid aqueous battery. J. Mater. Chem. A **6**(32), 15762-15770 (2018). https://doi.org/10.1039/C8TA04930K

[S4]Z. Hou, X. Li, J. Liang, Y. Zhu, Y. Qian, An aqueous rechargeable sodium ion battery based on a $NaMnO_2$–$NaTi_2(PO_4)_3$ hybrid system for stationary energy storage. J. Mater. Chem. A **3**(4), 1400-1404 (2015). https://doi.org/10.1039/C4TA06018K

[S5]G. Pang, P. Nie, C. Yuan, L. Shen, X. Zhang, J. Zhu, B. Ding, Enhanced performance of aqueous sodium-ion batteries using electrodes based on the $NaTi_2(PO_4)_3$/MWNTs–$Na_{0.44}MnO_2$ system. Energy Technol. **2**(8), 705-712 (2014). https://doi.org/10.1002/ente.201402045

[S6]Q. Yang, S. Cui, Y. Ge, Z. Tang, Z. Liu, H. Li, N. Li, H. Zhang, J. Liang, C. Zhi, Porous single-crystal $NaTi_2(PO_4)_3$ via liquid transformation of TiO_2 nanosheets for flexible aqueous Na-ion capacitor. Nano Energy **50**, 623-631 (2018). https://doi.org/10.1016/j.nanoen.2018.06.017

[S7]B. Zhao, Q. Wang, S. Zhang, C. Deng, Self-assembled wafer-like porous $NaTi_2(PO_4)_3$ decorated with hierarchical carbon as a high-rate anode for aqueous rechargeable sodium batteries. J. Mater. Chem. A **3**(22), 12089-12096 (2015). https://doi.org/10.1039/C5TA02568K

[S8]G. Pang, C. Yuan, P. Nie, B. Ding, J. Zhu, X. Zhang, Synthesis of nasicon-type structured $NaTi_2(PO_4)_3$–graphene nanocomposite as an anode for aqueous rechargeable Na-ion batteries. Nanoscale **6**(12), 6328-6334 (2014). https://doi.org/10.1039/C3NR06730K

[S9]Z. Liu, Y. An, G. Pang, S. Dong, C. Xu, C. Mi, X. Zhang, Tin modified $NaTi_2(PO_4)_3$ as an anode material for aqueous sodium ion batteries. Chem. Eng. J. **353**, 814-823 (2018). https://doi.org/10.1016/j.cej.2018.07.159

[S10] Z. Li, D. Young, K. Xiang, W.C. Carter, Y.-M. Chiang, Towards high power high energy aqueous sodium-ion batteries: The $NaTi_2(PO_4)_3$/$Na_{0.44}MnO_2$ system. Adv. Energy Mater. **3**(3), 290-294 (2013). https://doi.org/10.1002/aenm.201200598
